# A 18p11.23-p11.31 microduplication in a boy with psychomotor delay, cerebellar vermis hypoplasia, chorioretinal coloboma, deafness and GH deficiency

**DOI:** 10.1186/s13039-016-0298-9

**Published:** 2016-12-03

**Authors:** Mara Giordano, Valentina Muratore, Deepak Babu, Cristina Meazza, Mauro Bozzola

**Affiliations:** 1 Laboratory of Genetics, Department of Health Sciences, University of Eastern Piedmont, Novara, Italy; 2 Internal Medicine and Therapeutics Department, Pediatric and Adolescent Unit, University of Pavia, Fondazione IRCCS Policlinico San Matteo, Pavia, Italy

**Keywords:** 18p11.31-p11.23 microduplication, Growth hormone deficiency, Hypoplasia of the cerebellar vermis, Chorioretinal coloboma, Deafness

## Abstract

**Background:**

Rearrangements involving the short arm of chromosome 18 have been extensively described. Here we report a microduplication of 320.5–431.5 Kb at 18p11.31-p11.23 in a 10 year-old boy.

**Case presentation:**

In a 10 year-old boy with moderate psychomotor delay, hypoplasia of the cerebellar vermis, chorioretinal coloboma, deafness and growth hormone deficiency (GHD), an interstitial microduplication at 18p11.31-p11.23 was identified by array-CGH. This maternally inherited microduplication, encompasses three genes, namely *ARHGAP28*, *LINC00668* and *LAMA1* (a gene involved in cerebellum and retinal development).

**Conclusions:**

The genotype-phenotype is discussed with particular attention to the *LAMA1* gene, although it is difficult, as in many other similar situations, to assess the causality of the detected duplication in the absence of further studies aiming to explore the presence of co-occurring variants that could explain the incomplete penetrance.

**Electronic supplementary material:**

The online version of this article (doi:10.1186/s13039-016-0298-9) contains supplementary material, which is available to authorized users.

## Background

Rearrangements involving the short arm of chromosome 18 have been extensively described in the literature [1]. In most cases, patients carrying deletions of the whole arm (18p-syndrome), with breakpoints close to the centromeric region have been reported. The primary features of this condition include a cognitive impairment of varying severity, speech delay, short stature, holoprosencephaly, ptosis, pectus excavatum and dysmorphic features [2, 3]. Pituitary hormone deficiencies have also been reported [4, 5]. Conversely, 18p duplications are rare events in cytogenetic findings and only few cases are represented by pure duplications [1], being associated in most of the patients with 18q s or resulting from the unbalanced segregation of parental reciprocal translocations. For these patients, the phenotypic features could be the result of the excess of chromosome 18p material along with the imbalance of other chromosome regions.

Interestingly, there have been no reports of large interstitial duplication of 18p; nevertheless, with the introduction of microarrays some microduplications, with a likely pathogenic effect, have been identified. An 18p11.31-p11.32 duplication of 429.5 Kb, including four genes, among which the likely causative *EMILIN2*, has been identified in a family with porokeratosis of Mibelli [6]. In the same region, other authors [7] detected a larger overlapping 2.6 Mb microduplication involving at least nine genes in two siblings with variable levels of intellectual disability/developmental delay and behavioral difficulties. Finally, Kashevarova et. al [8] reported a microduplication of 351 kb at 18p11.32 in a boy with autism and dysmorphisms.

Here we report a microduplication of 320.5–431.5 Kb at 18p11.31-p11.23 identified through array comparative genomic hybridization (aCGH), not overlapping with the previously described one, encompassing three genes, namely *ARHGAP28*, *LINC00668* and *LAMA1* in a 10 year-old boy with moderate psychomotor delay, hypoplasia of the cerebellar vermis, chorioretinal coloboma, deafness and growth hormone deficiency (GHD). The functional role of laminin alpha 1 in cerebellum and retinal development suggests that the duplication of *LAMA1* may be responsible for at least some of the patient’s clinical features.

## Case presentation

A 7.5 year-old boy, the second child of non-consanguineous Caucasian healthy parents (father 33 years-old, with a height of 161.5 cm and mother 36 years-old, 166.1 cm) was referred to our Department because of short stature. His height was 110 cm (−2.25 standard deviation score (SDS), weight 17 Kg (body mass index (BMI) −4.78 SDS) and bone age 6.5 years. An intrauterine growth restriction was documented by ultrasound scan between the 20th and 22nd week of gestation. Labor was induced due to interruption of fetal development and fetal distress at 33 weeks. The birth weight was 1,450 g (−1.81 SDS), birth length 41 cm (−1.49 SDS) and head circumference 28 cm (−2.29 SDS). Apgar scores were 6 and 9 at 1 and 5 min. He required intubation and mechanical ventilation because of pneumothorax. He was discharged from the Neonatal Intensive Care Unit after 37 days with the diagnosis of chorioretinal coloboma. The patient’s family history was not significant for chromosome abnormalities, developmental delay or intellectual disability. His 3 year-old sister was healthy.

At 1 year of age, brain magnetic resonance image (MRI) showed lateral and third ventricle enlargement, stenosis of the aqueduct of Silvio and decrease dimensions of cerebellar vermis. These pathologic features were later confirmed by further MRI.

The patient had a history of significant developmental delay and intellectual disability. He sat at 13 months and walked at 2 years. He achieved language acquisition at 4 years and required speech therapist and psychologist care. At the age of 6 year the patient was diagnosed with bilateral mixed hypoacusis. At the age of 9.5 years, his intelligence quotient (IQ) was 49.

**Fig. 1:**
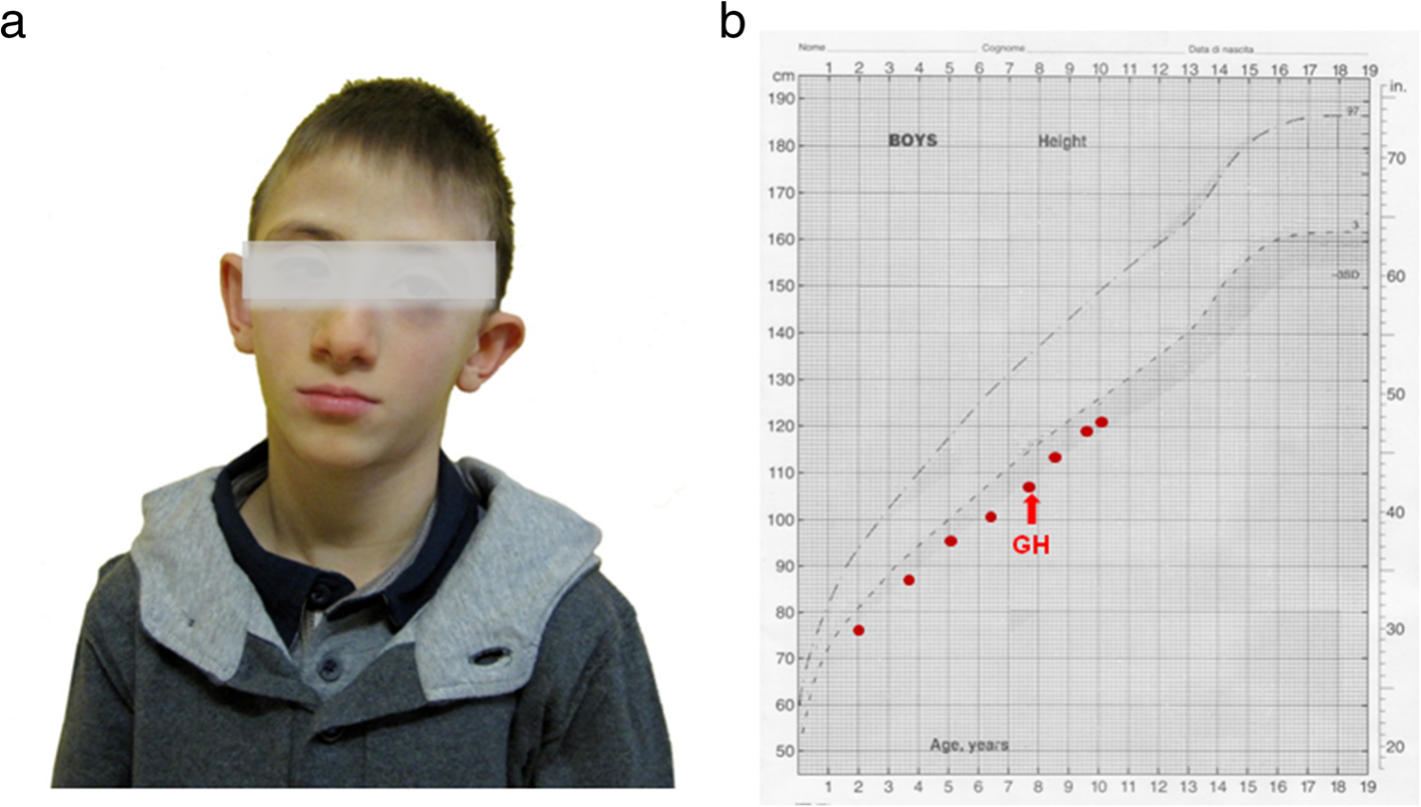
**a** Dismorphic features of the patient at 7.5 years of age. Microcephaly, triangular face, high forehead, protruding low set ears, micrognathia, esotropia, epicanthus, right palpebral ptosis are shown. **b** Height curve of the subject. The *arrow* indicates the start of GH substitutive therapy

He presented dysmorphic features including microcephaly, triangular face, high forehead, protruding low set ears, small teeth, micrognathia, esotropia, epicanthus, right palpebral ptosis and flat feet (Fig. 1a).

**Fig. 2:**
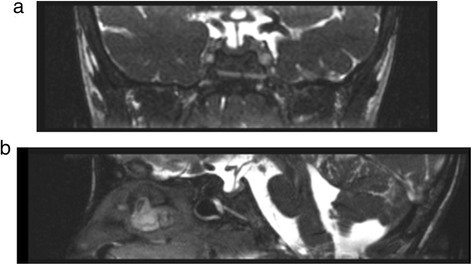
Magnetic resonance imaging of the hypothalamic-pituitary region. Flattened pituitary gland. **a** coronal view. **b** sagittal view

At the age of 7.5 years, he was investigated because of short stature. After exclusion of celiac disease, malabsorption, inflammatory bowel disease, hypothyroidism and other chronic diseases, GH response to two pharmacological stimuli (arginine and glucagon) revealed a GHD (GH peaks: 4.8 and 8.1 ng/ml, respectively; normal values >10 ng/ml). No other pituitary hormone deficiencies were found to be associated. MRI of the hypothalamic-pituitary region revealed a flattened pituitary gland (Fig. 2a, b). Substitutive GH therapy (0.21 mg/Kg subdivided into 6 weekly subcutaneous injections) was started and led to a satisfactory increase in height (Fig. 1b).

## Methods

### Array Comparative Genomic Hybridization (aCGH) analysis

DNA was extracted from whole blood of the patient by salting out method and the Concentration and purity was measured with the NanoDrop spectrophotometer (NanoDrop Technologies, Inc.). Array CGH was performed using the standard Agilent Human Genome G3 SurePrint 4 × 180 K Microarray (Agilent Technologies, California, USA), with median spacing of 13 Kb. Labeling and hybridization were carried out according to the manufacturer’s instruction (Agilent Technologies, California, USA).

The image of the array was acquired using the Agilent laser scanner G2565CA (Agilent Technologies, California, USA) and analyzed with the Agilent Feature Extraction software (v10.10.1.1.). Results were analyzed by Agilent Cytogenomics software (v2.5.8.11).

The presence of the duplication was confirmed in the patient and searched in his relatives through MLPA (Multiplex Ligation Probe Amplification) with a probe specifically designed within *LAMA1* (chr18:7014093–7014542; GRCh37/hg19) using the SALSA MLPA probemix P200-A1 Human DNA Reference-1 (MRC-Holland).

Copy number variations (CNVs) reported in the Database of Genomic Variants [9] were excluded from further analysis.

We performed a search in DECIPHER database [10] and ClinGen [11] to search for patients carrying similar micro-duplications.

### Sequencing

The *CDH7* and *LIG4* genes were screened for the presence of causative mutations by direct sequencing. Briefly, genomic DNA was amplified by polymerase chain reaction (PCR) using primers designed to specifically amplify the coding regions and the intron/exon boundaries of each gene (see Additional file 1) The PCR products were visualized on a 2% agarose gel and purified using ExoSAP-IT enzymatic PCR clean up system (Affimetrix, Santa Clara, CA). The purified products were, then, sequenced with the Big Dye Terminator kit (Applied Biosystems, Foster City, CA) and the automatic sequencer ABI PRISM 3100 Genetic Analyzer (Applied Biosystems, Foster City, CA).

## Results

At the age of 8 years, short stature, reduced head circumference, cognitive delay and facial dysmorphisms led to investigation of the *LIG4* gene involved in Dubowitz syndrome (OMIM #223370): no mutation was identified. Because of clinical features including chorioretinal coloboma, growth failure, developmental retardation and hearing loss, CHARGE syndrome (OMIM#214800) was suggested at the age of 9 years. Sequencing of the *CHD7*, whose mutation explains about 90% of cases of CHARGE syndrome, excluded this gene as the cause of the patient’s phenotype.

**Fig. 3:**
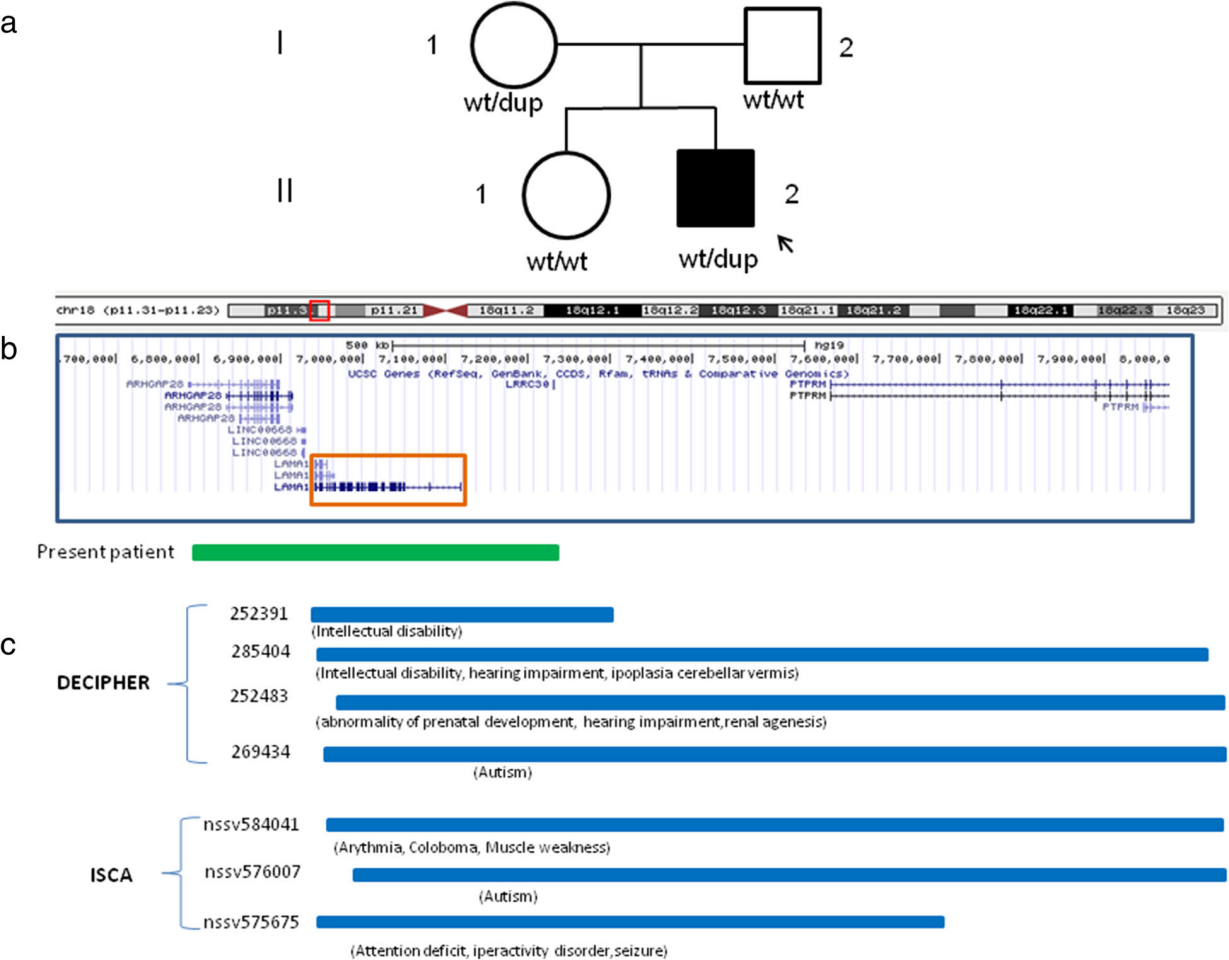
18p11.31-p11.23 duplication. **a** Pedigree of the family. The *arrow* indicates the proband. dup = duplication, wt = absence of the duplication. **b** Graphic representation of part of 18p11.23-p11.31 (corresponding to the region included in *red box* depicted in the chromosome 18 ideogram) with the genes included in this region. The duplication detected in our patient at 18p11.31-p11.23 18p11.23-p11.31 (6,813,085 × 2, 6,825,044–7,157,962 × 3, 7,193,872 × 2) -ranging from 332,9 to 380,7 Kb is represented by a *green rectangle* above the boxed region. Genomic positions refer to the Human Genome February 2009 assembly (GRCh37/hg19). **c** Schematic representation of the microduplications (smaller than 2.7 Mb) in this region reported in the DECIPHER and ISCA database. The ID of each patient is reported. The clinical features of the patients are reported in parentheses below the corresponding duplication as described in Decipher [10] and ClinGen [11]

The array-CGH performed at the age of 10 years showed the presence of an interstitial microduplication at 18p11.31-p11.23 (6,813,085 × 2, 6,825,044–7,157,962 × 3, 7,193,872 × 2) of 332,9–380,7 Kb (Fig. 3a). The same duplication, tested through MLPA, was present in the mother of the patient, but not in the father and in the older sister (Fig. 3b). Actually, the apparently healthy mother upon a closer evaluation revealed some mild clinical features of the son, such as partially hearing loss and mild micrognathia. She had a normal head size and her performance at school was within the normal range.

The duplicated region was examined using the human resource websites at NCBI [12] and the archive EnsEMBL [13]. This region contains 3 genes, encoding for a member of the Rho guanosine triphosphatases (*ARHGAP28*), the laminin subunit α1 (*LAMA1*) and a long intergenic non-coding RNA (*LINC00668*) of unknown function.

## Conclusions

Most microdeletions and microduplications encompass several genes with various functions, creating a challenge in understanding the role of the genes involved in the phenotype. Occasionally, however, these mutations encompass only few genes. Such cases are of special interest in contributing to the understanding of effects of dosage variations of the included genes.

Here we report a 18p micro-duplication including three genes in a patient with intellectual disability, deafness, GHD and other clinical features. The duplication was inherited from the unaffected mother. In contrast to microdeletions, the interpretation of microduplications often remains unsolved as the functional consequences of these alterations are not well understood. However, analyzing the role of the three genes included in the rearrangement, namely *ARHGAP28*, *LINC00668*, *LAMA1*, might help to elucidate, at least in part, the genotype-phenotype relationship.

The *ARHGAP28* encodes for a Rho GTPase activating protein (RhoGAP) that switches RhoA into its inactive form by stimulating the ability of Rho GTPase to hydrolyze GTP to GDP. The Rho GTPases are important in the regulation of reorganization of the actin during the formation of stress fibers. In particular it has been recently reported that in mice Arhgap28 might act as a negative regulator of RhoA, during the formation of actin stress fibers in cells of mesenchymal origin [14]. LINC00668 encodes for a long intergenic non coding RNA whose function has not been reported but similarly to the other non coding RNA it might be involved in the regulation of gene expression.

Among the genes included in the duplication, the best known is *LAMA1* as it has also been found mutated in humans and animal models. Laminins are a large family of heterotrimeric glycoproteins consisting of a α, β and γ chain present in the extracellular matrix, essential for the basement membrane assembly and critical for early embryonic development [15]. *LAMA1* encodes for the alpha-1 subunit of the Laminin-111 heterotrimer, formed by α1, β1 and γ1. Hypomorphic and null *LAMA1* mutations in mice and zebrafish cause defects in the retinal inner, limiting membrane development, abnormal retinal blood vessel formation and progressive cell loss from the inner nuclear and ganglion cell layers [16, 17]. In humans, *LAMA1* biallelic mutations have been identified in patients affected by cerebellar dysplasia with cysts, presenting defects in the retina development, including retinal dystrophy, absent pigment and atrophy [18]. However, both human and mice phenotypes are determined by inactivating mutations with the consequent absence of a functional product during retinal and cerebellum development, whereas here we detected a duplication of the gene containing region.

We can speculate that an excess of *LAMA1* may be involved in the cerebellum and retinal defects observed in our patient by perturbing the interaction with the other subunits in Laminin-111 formation, thus contributing to the cerebellar vermis hypoplasia and retinal coloboma. Alternatively, the long intergenic non-coding RNA (*LINC00668*) located downstream of the gene, might be involved in *LAMA1* transcription regulation and the increased level of this non-coding transcript might alter the local chromatin structure and consequently affect transcription of the nearby genes [19].

Regarding the hearing loss, it is intriguing that laminins have been detected at multiple sites in human and rodent inner ears [20, 21]. Furthermore, the *dy* mouse, a mutant model for congenital muscular dystrophy defective for laminin-α2, also presents hearing loss [22], thus confirming the pleiotropic effect of laminins and reinforcing the evidence of their involvement in deafness.

Although duplications encompassing *LAMA1* are not reported in the literature, seven patients with similar partial or complete overlapping imbalances, including this gene, are present in the DECIPHER [10] and ISCA Databases [11] (Fig. 3c). Remarkably, some of these patients present clinical features resembling those observed in our patient, such as hearing impairment in #285404 and #252483 and hearing impairment in association with hypoplasia of the cerebellar vermis in #285404 or Coloboma in nssv584041 and intellectual disability in #252391. In all these patients, the minimal critical region contains *LAMA1* (Fig. 3c). However, against the involvement of this duplication in the clinical phenotype is the observation that duplications including *LAMA1* also represent rare CNVs as reported in the DGV [9].

In any case, the interpretation of the role of this duplication remains challenging, also considering the paucity of similar rearrangements on 18p described in literature. To the best of our knowledge, there are only three interstitial submicroscopic duplications reported involving 18p and none of them overlaps with the one described here [6–8]. In all of these cases, the microduplication was either inherited from a healthy parent [7, 8], as in the here presented case, or it was present in unaffected family members [6], in accordance with a dominant model of inheritance with incomplete penetrance. The variability in the phenotype of patients carrying the same unbalances, ranging from severe disorders to healthy phenotype, might be explained with the presence of additional anomalies in the affected patients. This additional hit has been demonstrated in cases in which the aCGH revealed the presence of a second rearrangement that might operate through an additive model or by modifying the phenotype [23]. Further studies are required to establish the pathogenicity of this variation. Similarly to other imbalances with reduced penetrance and variable expressivity, it can be suspected that there might be other co-occurring variations not detectable with conventional aCGH. The complex phenotype of the here described patient could be the consequence of different and independent genetic defects and for example GHD could be determined by a causative mutation in some other gene.

## Additional file

Additional file 1: Supplementary Methods. (DOCX 22 kb)

## Abbreviations

aCGH: Array comparative genomic hybridization
GHD: Growth hormone deficiency
BMI: Body mass index
SDS: Standard deviation score
MRI: Magnetic resonance image
IQ: Intelligence quotient
CNVs: Copy number variations

## References

[1] Hasi-Zogaj M, Sebold C, Heard P, Carter E, Soileau B, Hill A, Rupert D, Perry B, Atkinson S, O’Donnell L, Gelfond J, Lancaster J, Fox PT, Hale DE, Cody JD (2015). A review of 18p deletions. Am J Med Genet C Semin Med Genet.

[2] Taine L, Goizet C, Wen ZQ, Chateil JF, Battin J, Saura R, Lacombe D (1997). 18p monosomy with midline defects and a de novo satellite identified by FISH. Ann Genet.

[3] Tonk V, Krishna J (1997). Case report: denovo inherited 18p deletion in a mother-fetus pair with extremely variable expression, confirmed by fluorescence in situ hybridization (FISH) analysis. Eur J Obstet Gynecol Reprod Biol.

[4] Artman HG, Morris CA, Stock AD (1992). 18p- syndrome and hypopituitarism. J Med Genet.

[5] Portnoï MF, Gruchy N, Marlin S, Finkel L, Denoyelle F, Dubourg C, Odent S, Siffroi JP, Le Bouc Y, Houang M (2007). Midline defects in deletion 18p syndrome: clinical and molecular characterization of three patients. Clin Dysmorphol.

[6] Occella C, Bleidl D, Nozza P, Mascelli S, Raso A, Gimelli G, Gimelli S, Tassano E (2013). Identification of an interstitial 18p11.32-p11.31 duplication including the *EMILIN2* gene in a family with porokeratosis of Mibelli. PLoS One.

[7] Balasubramanian M, Sithambaram S, Smith K (2016). Inherited duplication of the short arm of chromosome 18p11.32-p11.31 associated with developmental delay/intellectual disability. Clin Dysmorphol.

[8] Kashevarova AA, Nazarenko LP, Skryabin NA, Salyukova OA, Chechetkina NN, Tolmacheva EN, Sazhenova EA, Magini P, Graziano C, Romeo G, Kučinskas V, Lebedev IN (2014). Array CGH analysis of a cohort of Russian patients with intellectual disability. Gene.

[9] Database of Genomic Variants: http://dgv.tcag.ca/dgv/app/home. Accessed 1 Sept 2016.

[10] DECIPHER v9.10: Mapping the clinical genome: https://decipher.sanger.ac.uk. Accessed 1 Sept 2016.

[11] Clin Gen-Clinical Genome Resource: https://www.clinicalgenome.org. Accessed 1 Sept 2016.

[12] NCBI Human Genome Resources: http://www.ncbi.nlm.nih.gov/projects/genome/guide/human. Accessed 1 Sept 2016.

[13] EnsEMBL Ensemble Genome Broser 85: http://www.ensembl.org/index.html. Accessed 1 Sept 2016.

[14] Yeung CY, Taylor SH, Garva R, Holmes DF, Zeef LA, Soininen R, Boot-Handford RP, Kadler KE (2014). Arhgap28 is a RhoGAP that inactivates RhoA and downregulates stress fibers. PLoS One.

[15] Domogatskaya A, Rodin S, Tryggvason K (2012). Functional diversity of laminins. Annu Rev Cell Dev Biol.

[16] Edwards MM, Mammadova-Bach E, Alpy F, Klein A, Hicks WL, Roux M, Simon-Assmann P, Smith RS, Orend G, Wu J, Peachey NS, Naggert JK, Lefebvre O, Nishina PM (2010). Mutations in Lama1 disrupt retinal vascular development and inner limiting membrane formation. J Biol Chem.

[17] Edwards MM, McLeod DS, Grebe R, Heng C, Lefebvre O, Lutty GA (2011). Lama1 mutations lead to vitreoretinal blood vessel formation, persistence of fetal vasculature, and epiretinal membrane formation in mice. BMC Dev Biol.

[18] Aldinger KA, Mosca SJ, Tétreault M, Dempsey JC, Ishak GE, Hartley T, Phelps IG, Lamont RE, O’Day DR, Basel D, Gripp KW, Baker L, Stephan MJ, Bernier FP, Boycott KM, Majewski J (2014). Mutations in LAMA1 cause cerebellar dysplasia and cysts with and without retinal dystrophy. Am J Hum Genet.

[19] Li X, Wu Z, Fu X, Han W (2014). IncRNAs: insights into their function and mechanics in underlying disorders. Mutat Res Rev Mutat Res.

[20] Yamashita H, Bagger-Sjoback D, Wersall J (1991). The presence of laminin in the fetal human inner ear. Eur Arch Otorhinolaryngol.

[21] Rodgers L, Barritt JH, Miner D, Cosgrove D (2001). The laminins in the murine inner ear: developmental transitions and expressions in cochlear basement membranes. Hear Res.

[22] Pillers DA, Kempton JB, Duncan NM, Pang J, Dwinnell SJ, Trune DR (2002). Hearing loss in the laminin-deficient dy mouse model of congenital muscular dystrophy. Mol Genet Metab.

[23] Girirajan S, Rosenfeld JA, Cooper GM, Antonacci F, Siswara P, Itsara A, Vives L, Walsh T, McCarthy SE, Baker C, Mefford HC, Kidd JM, Browning SR, Browning BL, Dickel DE, Levy DL, Ballif BC, Platky K, Farber DM, Gowans GC, Wetherbee JJ, Asamoah A, Weaver DD, Mark PR, Dickerson J, Garg BP, Ellingwood SA, Smith R, Banks VC, Smith W, McDonald MT, Hoo JJ, French BN, Hudson C, Johnson JP, Ozmore JR, Moeschler JB, Surti U, Escobar LF, El-Khechen D, Gorski JL, Kussmann J, Salbert B, Lacassie Y, Biser A, McDonald-McGinn DM, Zackai EH, Deardorff MA, Shaikh TH, Haan E, Friend KL, Fichera M, Romano C, Gécz J, DeLisi LE, Sebat J, King MC, Shaffer LG, Eichler EE (2010). A recurrent 16p12.1 microdeletion supports a two-hit model for severe developmental delay. Nat Genet.

