# Supplementary material for: A 18p11.23-p11.31 microduplication in a boy with psychomotor delay, cerebellar vermis hypoplasia, chorioretinal coloboma, deafness and GH deficiency
Source: Mol Cytogenet. 2016 Dec 3;9:89. doi: 10.1186/s13039-016-0298-9 (PMC5135744; doi:10.1186/s13039-016-0298-9)
Supplement: Additional file 1: — Supplementary Methods. (DOCX 22 kb) [file 13039_2016_298_MOESM1_ESM.docx]

**Genetic analysis of *CHD7* and *LIG4***

Amplification of the *CHD7* (NM_017780) coding region was performed with previously described primers [1] designed at exon-intron boundaries of exons 2–38 (Table S1). Overlapping PCR fragments (NM_002312) corresponding to the two exons of the *LIG4* gene were amplified with previously published primers (Table S2) [2]. The PCR conditions were the same as those already published for the two genes in the corresponding papers apart the thermal cycle condition. For both the sets of primes a touchdown protocol from 65°C to 55°C was applied. Briefly, the annealing temperature was programmed at 65°C for the first 20 cycles. Then, the annealing temperature was lowered in 0.5-increments every cycle until 55°C, at which 25 final cycles were performed (total of 45 cycles).

**REFERENCES**

**1.** Lalani SR, Safiullah AM, Fernbach SD, Harutyunyan KG, Thaller C, Peterson LE, McPherson JD, Gibbs RA, White LD, Hefner M, Davenport SL, Graham JM, Bacino CA, Glass NL, Towbin JA, Craigen WJ, Neish SR, Lin AE, Belmont JW.. Spectrum of CHD7 mutations in 110 individuals with CHARGE syndrome and genotype-phenotype correlation. Am J Hum Genet. 2006;78:303-14.

**2.** O'Driscoll M, Cerosaletti KM, Girard PM, Dai Y, Stumm M, Kysela B, Hirsch B, Gennery A, Palmer SE, Seidel J, Gatti RA, Varon R, Oettinger MA, Neitzel H, Jeggo PA, Concannon P.. DNA ligase IV mutations identified in patients exhibiting developmental delay and immunodeficiency. Mol Cell. 2001;8:1175-85.

Overlapping fragments

of the LIG4 gene were amplified by PCR using primers

published previously [O’Driscoll et al., 2001].

Overlapping fragments

of the LIG4 gene were amplified by PCR using primers

published previously [O’Driscoll et al., 2001].

Overlapping fragments

of the LIG4 gene were amplified by PCR using primers

published previously [O’Driscoll et al., 2001].

**Table S1: *CHD7* primer sequences.**

| **Exon** | **Forward (5’-3’)** | **Reverse (5’-3’)** |  |  |  |
| --- | --- | --- | --- | --- | --- |
| 2a | ttcaggtaccagaccagatacg | tacggactgattcattggagt |  |  |  |
| 2b | accagatacgagccccctac | acagcattggggtatcttgg |  |  |  |
| 2c | ggcagtattctcgatatccttacag | atcagtcgttcctggattgc |  |  |  |
| 2d | atgcagcagtctcgtccatt | acagggagattgatgcctga |  |  |  |
| 3 | gaaacatcagccactaactttca | cccctcatttcataggctgta |  |  |  |
| 4 | gccaatatgtatggatttatcagttg | caagataggggaggtcttgtg |  |  |  |
| 5 | gccactgtcttgggtttttg | ccaacattaggtggatgttcc |  |  |  |
| 6 | gtggtagcaaaggggaatga | tgggttaataaattagacaggattaga |  |  |  |
| 7 | tgtgctcatatgggagtaaatca | ccaaagtttcttaatttcccaac |  |  |  |
| 8 | tgttgctcagcagccttaat | atgcaagttgacagcaccaa |  |  |  |
| 9 | ttttatattgctgtgacccaaaa | gaccaggtctaggattctacca |  |  |  |
| 10 | gagcatgcttttccttaatgtg | ctccctggaactctccgatt |  |  |  |
| 11 | tggtaagaattggctgatgg | cacaaatgcatacccaaagg |  |  |  |
| 12 | agcctttgggtatgcatttg | ccttcccaagtcaccaagac |  |  |  |
| 13 | gagatctccaaagggataaatacg | gcatcaaattctgagcaacg |  |  |  |
| 14 | tgcctgattcctatactttgcat | ggttctgtgtactaggtgggaaa |  |  |  |
| 15 | cactgggctttgaaaaatgaa | caccatgaaatccccagtct |  |  |  |
| 16 | cagtttgcaatgggttttga | ttccacttttaggtggactgc |  |  |  |
| 17 | cgccaataaaccctatttgct | ctgagtgacgacgcaacatt |  |  |  |
| 18 | aggactccaccttcatgaccta | ttaaataaaggaaagtgccagaa |  |  |  |
| 19 | ggtttactctctttgagaaaaatgc | cccaatgcatcttgtaagca |  |  |  |
| 20, 21 | cggagcaaatacataaacaaaa | ggggtgtcacacaaattcaa |  |  |  |
| 22 | caacgctggtacctgacttaaa | acgaggcagggaaatatgg |  |  |  |
| 23 | gcctcgtgcattaagctctc | cctccaaatctgcgagttct |  |  |  |
| 24 | aggatgatggatgaacagca | gaaggttctgaaacacaatcca |  |  |  |
| 25 | cccaccatgctcagatgttt | gccaagagtcctttggaact |  |  |  |
| 26 | gttgtggcagtgctgtgatt | tgtgtactgcagggtaagaactg |  |  |  |
| 27, 28 | aaaagtagcactgggcagatt | gaaataaggtgttaagacactgctg |  |  |  |
| 29 | ccctttcccacactgtcatt | gagcctttctttggtggtca |  |  |  |
| 30 | ccacccccaaataactacca | tctgtaacacagaagggctca |  |  |  |
| 31a | gcaacaaagttctatacaaaaagacg | tgagcagatgagatgactgga |  |  |  |
| 31b | caaaacagaggggcaggtaa | ctcgtgaaaaagagcaggtg |  |  |  |
| 32 | tgcccaataccattctagcc | tctttgggctttttcagatca |  |  |  |
| 33 | tttgcatcttgatggatgtatt | gcaaggccagtgaaatataagc |  |  |  |
| 34 | aagccagcccatatagcagt | ggaggaagctggctttcata |  |  |  |
| 35 | ttgaaataggacattgtcagagg | attcaaggaaaaggcagagg |  |  |  |
| 36 | tctgacagttctctttggcatt | gaaggcaggataaaacacttcc |  |  |  |
| 37 | gaatgcgtgtgtgcgtgtat | ggccaacagaaaataagaaagac |  |  |  |
| 38a | atttggaatggcaggttcac | tcaggtccattccagcaaac |  |  |  |
| 38b | gaagaggaagaaggcccaaa | tcctggaggtagaaacatgg |  |  |  |
| 38c | tgactctgcgaatggatctg | tgaacattaactgtgagtgtaaacag |  |  |  |

**Table S2: *LIG4* Primer sequences.**

| **Exon** | **Forward (5’-3’)** | **Reverse (5’-3’)** |
| --- | --- | --- |
| 1 | gtagactgcgccgcattag | tctacatcattcctctgccc |
| 2.1 | ctcagtgatctttgtaagatc | gtaaacatcttggcttcaacac |
| 2.2 | ggagatgctggagactttgc | ttataggccatcatctcacca |
| 2.3 | cccattcattcataatgcattc | aaaggcttccaatacttggc |
| 2.4 | ggctgcaccatgaaagaact | catgcaggcttgacaacatc |
| 2.5 | gtacaaatccaggcccagac | catgagacactccctcagc |
| 2.6 | agcttcggtttcatggagc | tctttcttggctttgggcta |
